# Age- and sex-based changes in spike protein antibody status after SARS-CoV-2 vaccination and effect of past-infection in healthcare workers in Osaka

**DOI:** 10.1186/s12879-022-07695-7

**Published:** 2022-08-26

**Authors:** Shiro Hoshida, Nobuyuki Koeda, Hideki Hattori, Masahiro Tanaka, Ichiro Tanaka, Hiroyuki Fukui, Junya Fujita, Yo Sasaki, Shigeyuki Tamura

**Affiliations:** Department of Clinical Practice, Yao Municipal Hospital, 1-3-1 Ryuge-cho, Yao, Osaka 581-0069 Japan

**Keywords:** Age group, Nucleocapsid protein antibody, SARS-CoV-2, Sex, Spike protein antibody

## Abstract

**Objective:**

We aimed to compare the changes in SARS-CoV-2 spike protein antibody titres based on age group and sex using paired blood sampling after vaccination in association with the presence of nucleocapsid protein antibody.

**Methods:**

All participants were healthcare workers at Yao Municipal Hospital in Osaka who voluntarily provided peripheral blood samples (n = 636, men/women 151/485, mean age 45 years). We investigated the serial changes in SARS-CoV-2 spike protein antibody titres at 1 and 7 months after the second vaccination regarding their relationship with sex and age group. At 7 months, we also examined anti-nucleocapsid assays. Antibody titres were shown as logarithmic values and the differences were assessed using a paired or unpaired student’s t-test as appropriate.

**Results:**

Among participants younger than 30 years, the antibody titres of spike protein were significantly higher in women one (p = 0.005) and seven (p = 0.038) months after vaccination. However, among those aged 30–49 years, the antibody titres were not different between the sexes at either follow-up time point. In contrast, among those aged 50–59 years, between-sex differences in antibody titres were observed only at 7 months, which was associated with a significant reduction in men. A significant negative correlation was observed between the antibody titres for spike protein at both time points in participants with positive nucleocapsid protein antibody at 7 months (r = − 0.467, p = 0.043), although a significant positive correlation was observed in those with negative results (r = 0.645, p < 0.001),

**Conclusions:**

Between-sex differences in SARS-CoV-2 spike protein antibody titres by paired blood sampling at different time points after vaccination depended on age group. The presence of nucleocapsid protein antibody was associated with changes in spike protein antibody titres after vaccination.

**Supplementary Information:**

The online version contains supplementary material available at 10.1186/s12879-022-07695-7.

## Introduction

Since immunity against the severe acute respiratory syndrome coronavirus 2 (SARS-CoV-2) waned a few months after receipt of the second dose of vaccines [1, 2], checking the immune status after vaccination helps to determine the need for booster doses, and optimise vaccine intervals. Serologic testing of antibodies specific to SARS-CoV-2 spike protein or nucleocapsid protein is used to detect immune status. Real-world immunogenicity data describing antibody titres over time after vaccination are appearing [1]. Several reports found a significant correlation between antibody titres of spike protein and neutralising antibody titres [3, 4]. Furthermore, antibodies directed against the spike protein show potent antiviral activity and correlate with potential immunity [5]. Roche developed a serologic assay that measures antibodies against the receptor-binding domain of the spike protein, the target of vaccines in use, [6–8] and thus may aid in characterising the immune response to vaccines. Elecsys^®^ Anti-SARS-CoV-2 S immunoassay had higher sensitivity than several serological assays for detecting SARS-CoV-2 spike protein antibodies [7].

Recent studies provide conflicting evidence on the persistence of SARS-CoV-2 immunity induced by vaccines, but the data on paired blood sampling for antibody titres at two fixed time points for each participant are limited. Therefore, this study aimed to evaluate commercially available serological assays using paired samples from post-vaccination for SARS-CoV-2 with BNT162b2 [9] and compare the changes in antibody titres based on sex and age groups, and post-infection.

## Methods

### Participants

This cross-sectional study was conducted at the Yao Municipal Hospital in Osaka, Japan. All the participants were healthcare workers who had voluntarily provided peripheral blood samples for serologic assays one (May 2021) and 7 months (November 2021) after receiving the second vaccine dose (April 2021) (n = 636, men/women 151/485, mean age 45 years). In some populations (n = 357, men/women 100/257, mean age 48 years), peripheral blood samples for serological assays (late February to early March 2021) were also obtained before the first vaccine dose. All participants received the first dose of the BNT162b2 coronavirus disease vaccine between mid and late March 2021. This was followed by the original protocol of administering the second dose 3 weeks after the initial injection in all recipients. The participants included patient- and non-patient-facing staff members.

### Laboratory assays

All collected samples in March, May, and November 2021 were tested using the Roche Elecsys anti-spike protein of SARS-CoV-2 S, measuring total antibodies [6–8]. The Roche assay is an electrochemiluminescent immunoassay (ECLIA) that quantitatively detects antibodies to the SARS-CoV-2 spike protein receptor binding domain (including IgM and IgG) (Roche Diagnostics K.K., Japan). The assay results were interpreted using the manufacturers’ recommended specific thresholds. This assay is highly sensitive and specific [7]. Assays with good sensitivity have a lower percentage of false negatives. This assay has a measuring range of 0.40–250 U/mL (up to 2500 U/mL with on-board 1:10 dilution), with a concentration of < 0.80 U/mL considered negative and ≥ 0.80 U/mL considered positive.

To compare with an antibody test against spike protein of SARS-CoV-2, we also measured 636 serum samples collected in November 2021 with an anti-nucleocapsid protein of SARS-CoV-2 using an ECLIA kit obtained from Roche diagnostics K.K. (Japan). [10–14] According to the manufacturer’s instructions, the cut-off value for a positive SARS-CoV-2 antibody for nucleocapsid protein was deemed as 1 COI (cut-off index).

### Patient and public involvement

None of the participants were involved.

### Statistical analysis

The antibody titres for the spike protein of SARS-CoV-2 are shown as logarithmic values, and the data are expressed as mean ± standard deviation. Categorical variables are presented as frequencies and percentages. Differences in categorical variables between the groups were assessed using the chi-square test, while those in continuous variables were assessed using a paired or unpaired student’s t-test as appropriate.

## Results

### Related factors for antibody titres

Women were significantly younger than men. All blood samples at months 1 and 7 after vaccination showed positive results for antibody titres of the spike protein. To clarify the related factors, we examined the differences between younger (≤ 45 years) and older (> 45 years) age, sexes, and the participants with negative and positive results for antibody titres of nucleocapsid protein at month 7 of the second vaccination (Table 1). Younger people and women showed significantly higher antibody titres of spike protein, although no differences were observed in the reduced levels of antibody titres during the 6 months between the two groups. In participants showing positive results for antibody titres of nucleocapsid protein at month 7 as compared to those showing negative results, significantly higher antibody titres of spike protein were observed at both time points, along with a significant difference in the reduction level of the antibody titres during the 6 months. The number of participants with positive antibody titres for spike protein before vaccination was three (3/357, 0.9%), and all had positive antibody titres for nucleocapsid protein at month 7 after vaccination.

**Table 1:** Factors relating to the differences in SARS-CoV-2 antibody titres

	Age		p-value	Sex		p-value	N Ab		p-value
	Low (≤ 45 years)	High (> 45 years)		Men	Women		Negative	Positive	
	n = 324	n = 312		n = 151	n = 485		n = 617	n = 19	
Age, years	35.6 ± 7.8	54.1 ± 6.7	–	46.9 ± 13.4	44.0 ± 11.1	0.007	44.8 ± 11.8	40.4 ± 9.6	0.103
Men, n (%)	68 (21)	83 (27)	0.058	151 (100)	0 (0)	–	148 (24)	3 (16)	0.580
Log (S Ab) after 1 month of vaccination	3.11 ± 0.36	3.00 ± 0.35	< 0.001	3.00 ± 0.33	3.08 ± 0.36	0.029	3.04 ± 0.33	3.76 ± 0.55	< 0.001
Log (S Ab) after 7 months of vaccination	2.77 ± 0.39	2.65 ± 0.43	< 0.001	2.63 ± 0.41	2.74 ± 0.42	0.004	2.69 ± 0.37	3.64 ± 0.65	< 0.001
Delta log (S Ab)	− 0.34 ± 0.33	− 0.35 ± 0.36	0.638	− 0.37 ± 0.37	− 0.34 ± 0.34	0.257	− 0.35 ± 0.30	− 0.13 ± 1.03	0.004
N Ab positive after 7 months of vaccination, n (%)	12 (4)	7 (2)	0.198	3 (2)	16 (3)	0.289	0 (0)	19 (100)	–

Data are mean ± standard deviation or the numbers (percent)

SARS-CoV-2, severe acute respiratory syndrome coronavirus 2; N Ab, nucleocapsid protein antibody; S Ab, spike protein antibody

Antibody titres for spike protein are represented as logarithmic values [log (S Ab)]

Delta log (S Ab) represents changes in logarithmic values of S Ab from 1 to 7 months after vaccination

### Correlation between age and antibody titres

**Fig. 1:**
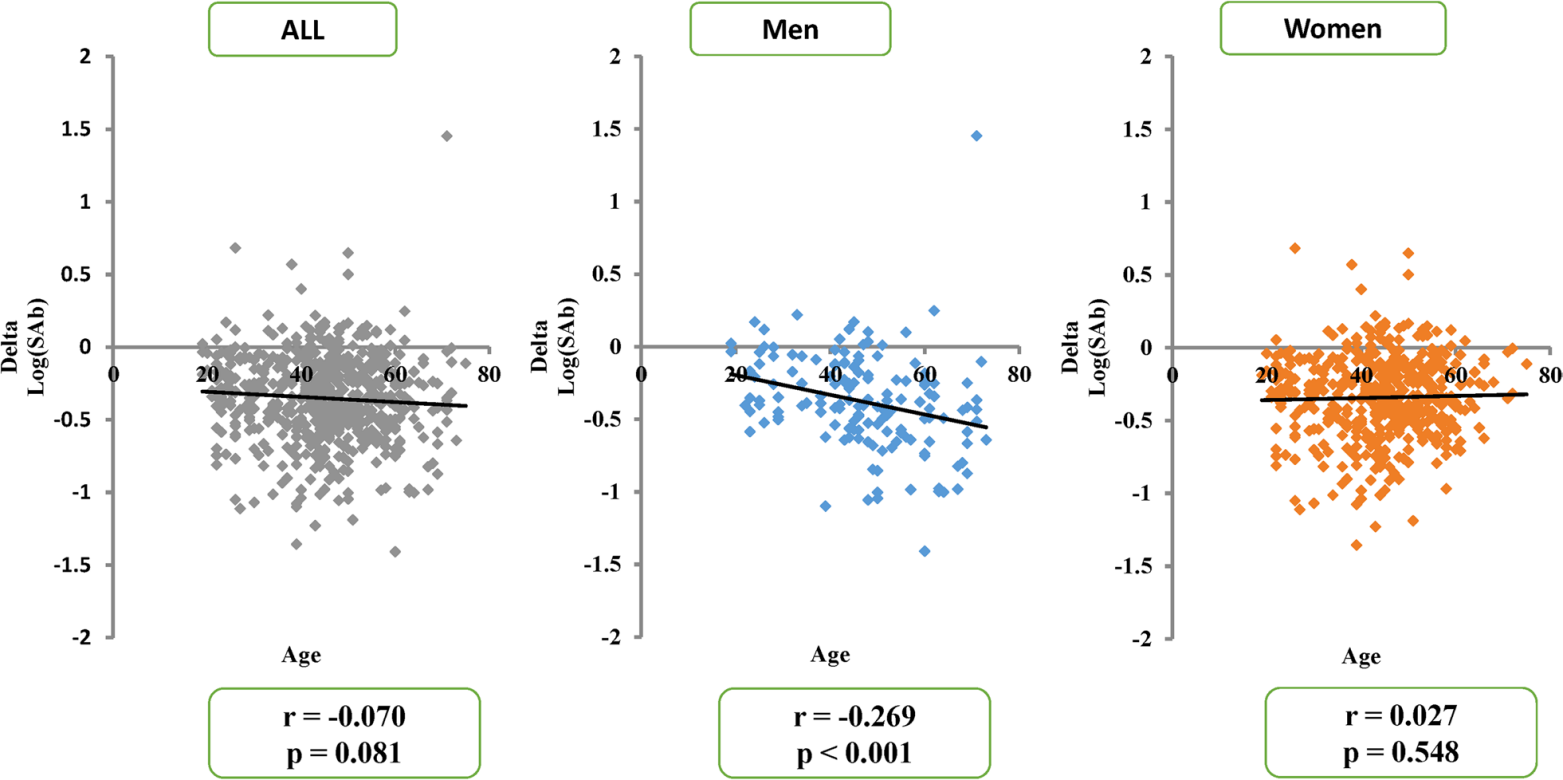
The correlation between age (horizontal axis) and the changes in logarithmic values of anti-spike antibody titres from one to seven months after vaccination [delta log (S Ab)] (vertical axis). The correlation was negatively significant only in men

The correlation between age and antibody titres was negatively significant at month 1 after vaccination (r = − 0.236, p < 0.001). Increasing age was significant for decreasing antibody titres at month 1 following dose 2 for both men (r = − 0.249, p = 0.002) and women (r = − 0.224, p < 0.001) (Additional file 1: Fig. S1). At month 7, this effect was only observed for men. When the correlation was examined between age and the reduction level of antibody titres at 1–7 months after vaccination, there was a significant negative correlation only in men (r = − 0.269, p < 0.001) (Fig. 1). Since the participants exhibiting positive antibody titres for nucleocapsid protein at month 7 showed higher antibody titres for spike protein for both time points and no significant reduction in antibody titres of spike protein was observed during the follow-up period, we excluded the data of those with positive antibody titres of nucleocapsid protein (Fig. 1).

### Differences in antibody titres between sexes in each age group

Among participants aged less than 30 years, antibody titres of spike protein were significantly higher in women at months 1 and 7 after vaccination (Table 2), although no differences were observed in mean age (25 ± 3 vs. 25 ± 3 years, p = 0.362) and in the reduction level of antibody titres during the follow-up period between sexes. However, among those aged 30–39 years or 40 to 49 years, antibody titres were not different between sexes at months 1 and 7 after vaccination, and there were no differences in mean age and the reduction level of antibody titres during the follow-up period. In contrast, among those aged 50–59 years, between-sex differences in antibody titres were observed only at month 7 and were associated with a significant reduction in antibody titres only in men (Table 2). There were no differences in mean age (53 ± 3 vs. 54 ± 3 years, p = 0.245) between sexes among this age group. Similar results were observed among participants aged ≥ 60 years, although the differences were insignificant.

**Table 2:** Differences in SARS-CoV-2 spike protein antibody titres based on sex and age group

Age	Sex		Log (S Ab)						Delta log (S Ab)		
	Men, n	Women, n	Month 1 (May 2021)		p-value	Month 7 (Nov 2021)		p-value	Men	Women	p-value
			Men	Women		Men	Women				
< 30 years	23	63	3.03 ± 0.24	3.25 ± 0.34	0.005	2.79 ± 0.24	2.95 ± 0.34	0.038	− 0.24 ± 0.23	− 0.30 ± 0.35	0.444
30–39 years	16	83	3.26 ± 0.48	3.13 ± 0.39	0.246	2.89 ± 0.37	2.72 ± 0.46	0.185	− 0.37 ± 0.32	− 0.40 ± 0.33	0.724
40–49 years	49	185	3.01 ± 0.26	3.05 ± 0.35	0.438	2.73 ± 0.42	2.69 ± 0.36	0.499	− 0.28 ± 0.36	− 0.36 ± 0.29	0.096
50–59 years	29	123	2.95 ± 0.25	3.03 ± 0.35	0.306	2.47 ± 0.34	2.76 ± 0.47	0.002	− 0.50 ± 0.29	− 0.28 ± 0.41	0.007
≥ 60 years	34	31	2.89 ± 0.40	2.89 ± 0.34	0.981	2.40 ± 0.39	2.57 ± 0.40	0.077	− 0.49 ± 0.47	− 0.32 ± 0.21	0.063

Data are the numbers, or logarithmic values of spike protein antibody titres [log (S Ab)] and are mean ± standard deviation

Delta log (S Ab) represents changes in logarithmic values of spike protein antibody titers [log (S Ab)] from 1 to 7 months after vaccination

SARS-CoV-2, severe acute respiratory syndrome coronavirus 2; S Ab, spike protein antibody

### Correlation between the antibody titres at months 1 and 7 after vaccination

**Fig. 2:**
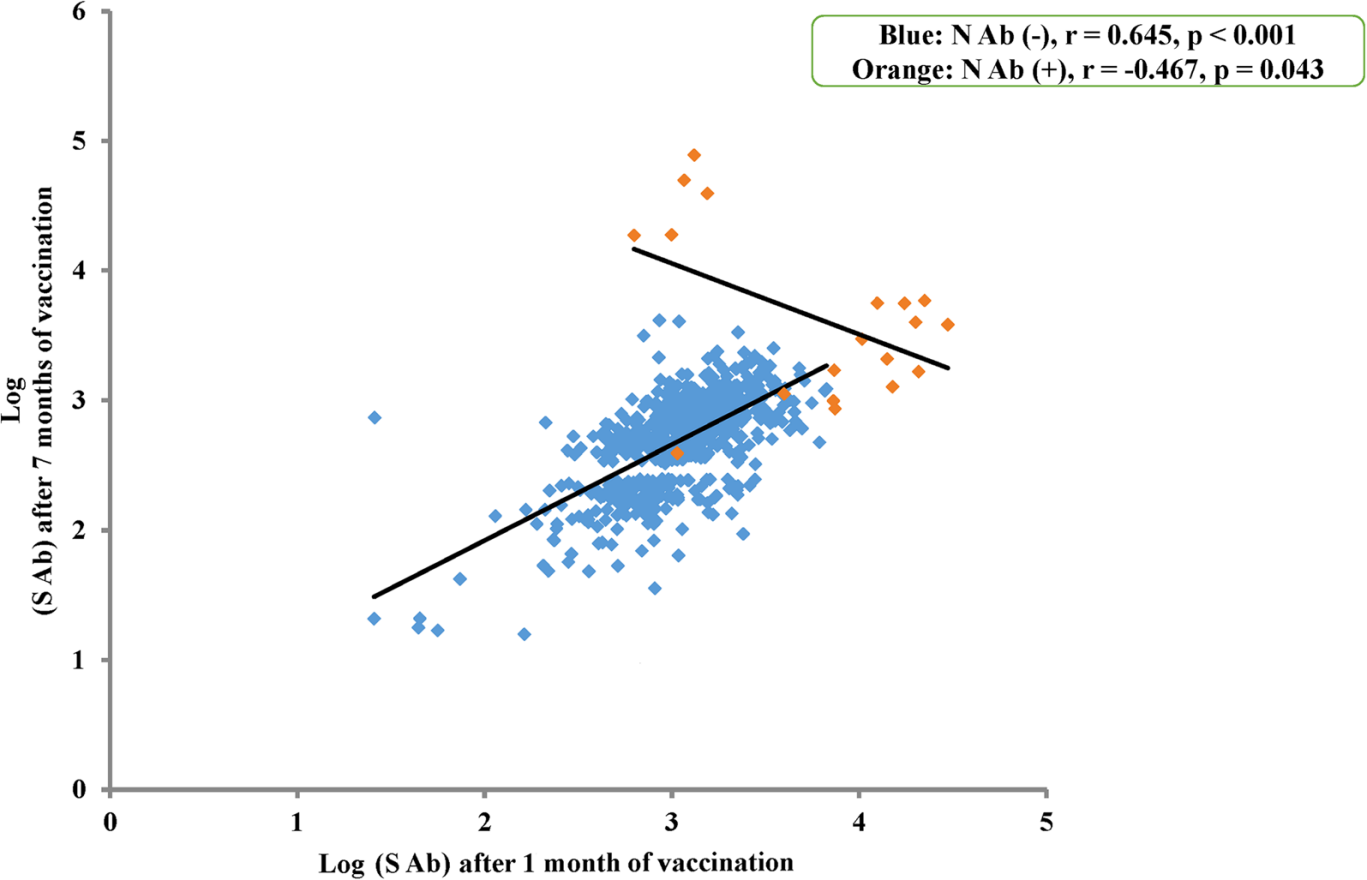
The correlation between the logarithmic values of anti-spike antibody titres [log (S Ab)] at one and seven months after vaccination. The correlation was positively significant in participants with negative antibody titres for nucleocapsid protein [N Ab (−)] at seven months after vaccination (n = 617, blue circles), but was negatively significant in those with positive antibody titres [N Ab (+)] (n = 19, orange circles)

A significant positive correlation was observed between the antibody titres of spike protein at months 1 and 7 after vaccination in men (r = 0.522, p < 0.001) and women (r = 0.638, p < 0.001) (Additional file 1: Fig. S2). The participants with positive nucleocapsid protein antibody at month 7 (n = 19), compared to those with negative results, had significantly higher antibody titres for spike protein at months 1 and 7, and a significantly smaller decrease in titres between the two collection timepoints. Therefore, the correlation between the antibody titres at months 1 and 7 after vaccination was examined separately in participants with and without positive nucleocapsid protein antibody (Fig. 2). In those with negative nucleocapsid protein antibody at month 7 after vaccination (n = 617), a significant positive correlation was observed between these antibody titres for spike protein, as shown in blue in Fig. 2 (r = 0.645, p < 0.001). However, a significant negative correlation was observed in participants with positive nucleocapsid protein antibody (n = 19), as shown in orange in Fig. 2 (r = − 0.467, p = 0.043). This was due to the presence of five participants whose antibody titres of spike protein were inversely higher at month 7 after vaccination than those at month 1. Among the 19 participants with positive nucleocapsid protein antibody at month 7, only six participants examined antibody titres of spike protein before vaccination (three positives and three negatives). In three participants with positive spike protein antibody before vaccination, antibody titres of spike protein were higher at month 1 after vaccination than those at month 7 or the other participants at month 1. In contrast, in two out of three participants without spike protein antibody before vaccination, antibody titres of spike protein were lower at month 1 after vaccination than those at month 7 after vaccination (results not shown).

## Discussion

Examination of commercially available serological assays is important to comprehend the immune response and the duration of post-vaccination protection. The antibody titres of SARS-CoV-2 spike protein were significantly higher in women at months 1 and 7 after vaccination among younger participants. A significant negative correlation was observed between age and changes in the antibody titres after vaccination only in men. Moreover, a significant positive correlation was observed between the antibody titres at months 1 and 7 after vaccination in participants with negative nucleocapsid protein, although a significant negative correlation was found in those with positive nucleocapsid protein antibody titres at month 7 after vaccination.

### Immunogenicity based on age and sex

Antibody responses after vaccination showed high interindividual variation in levels. Recent studies provide conflicting evidence on the persistence of SARS-CoV-2 immunity induced by mRNA vaccines [15–17]. Antibody kinetics showed differences in immunogenicity according to sex, age group, and coexisting conditions [1, 3, 15, 18]. In this study, we observed that between-sex differences in antibody titres for spike protein after vaccination differed according to age groups; only among participants younger than 30 years, the antibody titres of SARS-CoV-2 spike protein were significantly higher in women at months 1 and 7 after vaccination. This result could differ from the previous study [1] showing between-sex differences in all age groups due to the timetable for the measurement of antibody titres because we did not examine the peak level in each individual. The difference in the age groups could be another reason.

In our study, the correlation between age and antibody titres for spike protein was negatively significant at month 1 after vaccination in both sexes, as previously reported [19]. This finding is consistent with the results showing that larger differences are observed in antibody titres between younger and older age groups [1]. When the correlation was examined between age and the reduction levels of the antibody titres from month 1 to month 7 after vaccination, there was a significant correlation only in men. This finding was according to the between-sex difference in the antibody titres at month 7 after vaccination among participants aged 50 years or older. Immune maintenance capacity may be lower in men in the aged population.

### Infection affects the changes in antibody titres of spike protein after vaccination

Our findings using paired samples in each participant showed a positive correlation between the antibody titres for spike protein at months 1 and 7 after vaccination. However, in participants with positive nucleocapsid protein antibody at month 7 after vaccination, their antibody titres for spike protein at the two time points were high, and the correlation was significantly negative. Serologic testing of antibodies specific to SARS-CoV-2 nucleocapsid protein is used as a marker of a previous infection and may aid in surveying asymptomatic infection and assessing past SARS-CoV-2 infection prevalence. In the subset of the study population previously exposed to SARS-CoV-2 based on seropositivity for nucleocapsid antibodies, higher anti-spike IgG levels are already measured before the vaccine but no significant difference in anti-spike IgG levels from unexposed individuals is observed after vaccination [16]. We could not determine the time when the participants were positive for nucleocapsid protein antibodies at month 7 after vaccination infected SARS-CoV-2. Among the 19 participants with positive nucleocapsid protein antibody at month 7, only three participants had positive antibody titres of spike protein before vaccination and their antibody titres of spike protein were higher at month 1 than those at month 7 after vaccination or the other participants at month 1. The rest participants with positive nucleocapsid protein at month 7 (n = 16) may be infected after dose 1 and before blood collection at month 7. Although immunity against SARS-CoV-2 reduced after administration of the second dose of vaccine, [1, 2] the infection’s timing may affect antibody titres of spike protein after vaccination. At least five out of 19 participants with positive nucleocapsid protein at month 7 (Fig. 2) showed high antibody titres of spike protein at both time points, and their titres were inversely higher at month 7 after vaccination. Previous studies have reported that the antibody titres for spike protein are high [1, 19, 20] and their decay is slow [21, 22] in populations with a previous infection of SARS-CoV-2. These findings could be related to the fact that SARS-CoV-2 infection rates in those with nucleocapsid protein antibody positivity are low, compared to those with antibody-negativity [23, 24].

### Limitations

We did not examine the factors related to antibody titres, such as comorbidities, except for age and sex. [25] We did not evaluate neutralising antibody kinetics; the correlation between antibody titres and neutralising function would be dependent on the time after vaccination. [1] We did not examine the precise timing for the decrease in antibody titres after vaccination, although it may be brisk until 70–80 days after vaccination. [1] Data on previous positivity are available only on a limited number of subjects (anti-S titer before vaccination) and there is no information on swabs before and after vaccination (gold standard for the diagnosis of infection). The determination of positivity at month 7 after vaccination was made with antibodies to the nucleocapsid protein. This method has several critical issues, in particular a low sensitivity in subjects with asymptomatic or mild infection. The participants were mostly healthy, and therefore, may not represent the general population. Although the sample size was small, we presented new knowledge regarding the divergence in antibody titres for spike protein after vaccination, between the sexes and according to age group, and emphasized the optimisation of these assays for evaluating post-vaccination antibody status.

## Conclusions

Between-sex differences in antibody titres for SARS-CoV-2 spike protein after vaccination differed according to the age group. The correlation between age and reduced antibody titres for spike protein after vaccination was significant only in men. The antibody titres for spike protein after vaccination must be carefully evaluated in the absence or presence of SARS-CoV-2 infection.

### Strengths and limitations

Healthcare workers were enrolled in a fixed follow-up schedule with the paired data of antibody titres after vaccination.

We presented new knowledge regarding the divergence in antibody titres for spike protein after vaccination, between the sexes and according to age group.

The presence of nucleocapsid protein antibody was associated with changes in spike protein antibody titres after vaccination.

We did not evaluate neutralising antibody kinetics and did not examine the factors related to antibody titres, such as comorbidities, except for age and sex.

### What is already known on this topic

Antibody kinetics show differences in immunogenicity according to sex, age group, and coexisting conditions after vaccination for SARS-CoV-2.

Since immunity against SARS-CoV-2 waned a few months after receipt of the second dose, checking the immune status, post-vaccination, helps to determine the need for booster doses, and identify and optimise vaccine intervals.

We are beginning to see real-world immunogenicity data describing antibody kinetics over time after vaccination; however, data on paired blood sampling for antibody titres after vaccination in each participant are limited.

### What this study adds

Between-sex differences in SARS-CoV-2 spike protein antibody titres by paired blood sampling at different time points after vaccination depended on age group.

There was a significant negative correlation between age and the changes in antibody titres from 1 to 7 months after vaccination, in men alone.

The presence of nucleocapsid protein antibody was associated with changes in spike protein antibody titres after vaccination.

## Supplementary Information

**Additional file 1: Figure S1.** The correlation between age (horizontal axis) and logarithmic values of anti-spike antibody titres [log (S Ab)] at one month after vaccination (vertical axis) was negatively significant in men and women.

**Additional file 2: Figure S2.** The correlation between the logarithmic values of anti-spike antibody titres [log (S Ab)] at one and seven months after vaccination was positively significant, and no differences were observed between the sexes.

## Data Availability

The original dataset for this research included only age and sex along with antibody titres in a data repository. The datasets generated and/or analysed during the current study are not publicly available but are available from the corresponding author on reasonable request from the principal investigator.
