# Supplementary figures and images for: Age- and sex-based changes in spike protein antibody status after SARS-CoV-2 vaccination and effect of past-infection in healthcare workers in Osaka
Source: BMC Infect Dis. 2022 Aug 26;22:709. doi: 10.1186/s12879-022-07695-7 (PMC9412794; doi:10.1186/s12879-022-07695-7)

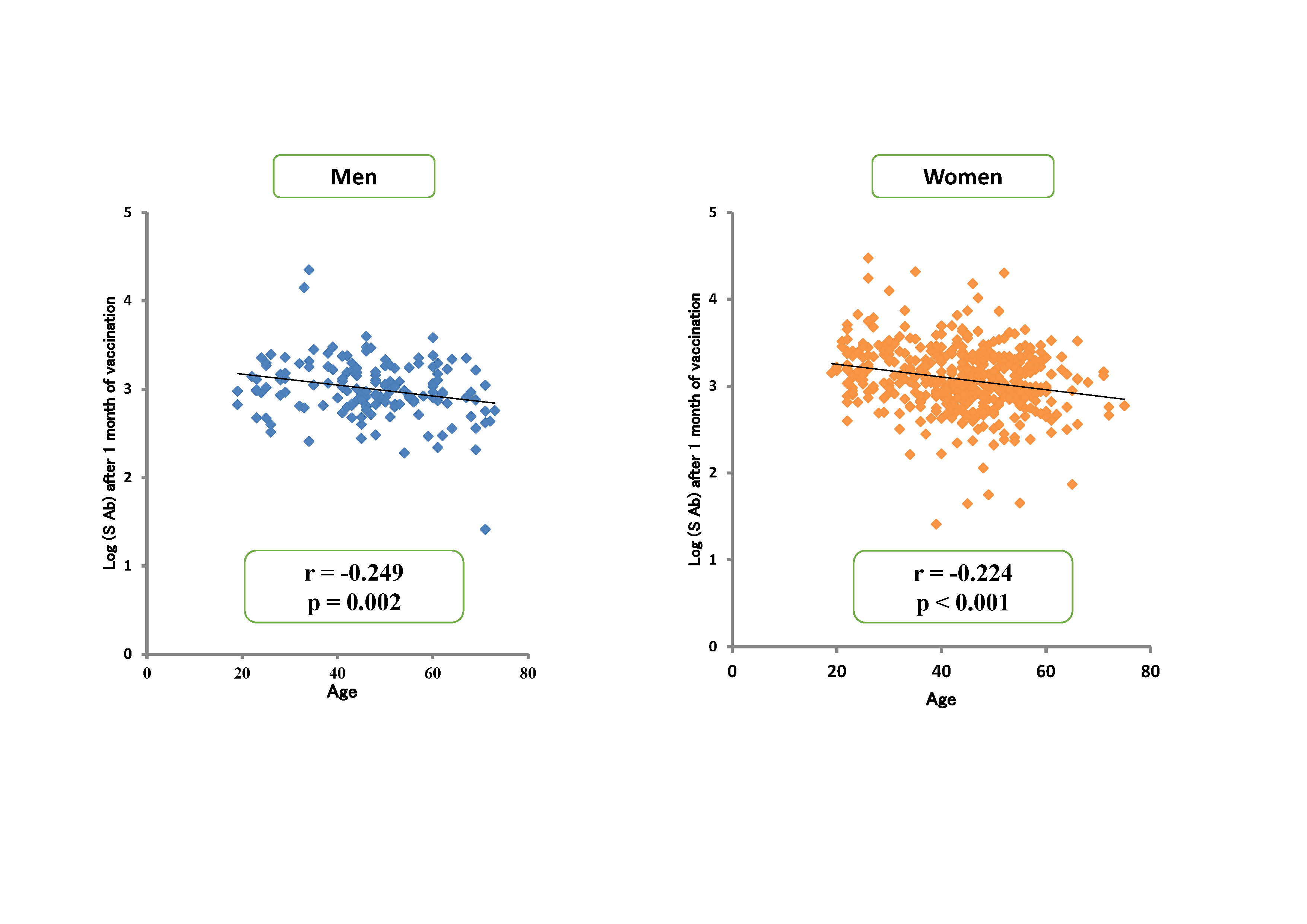

Supplement: Supplementary file 1 — Additional file 1: Figure S1. The correlation between age (horizontal axis) and logarithmic values of anti-spike antibody titres [log (S Ab)] at one month after vaccination (vertical axis) was negatively significant in men and women. [file 12879_2022_7695_MOESM1_ESM.tif]

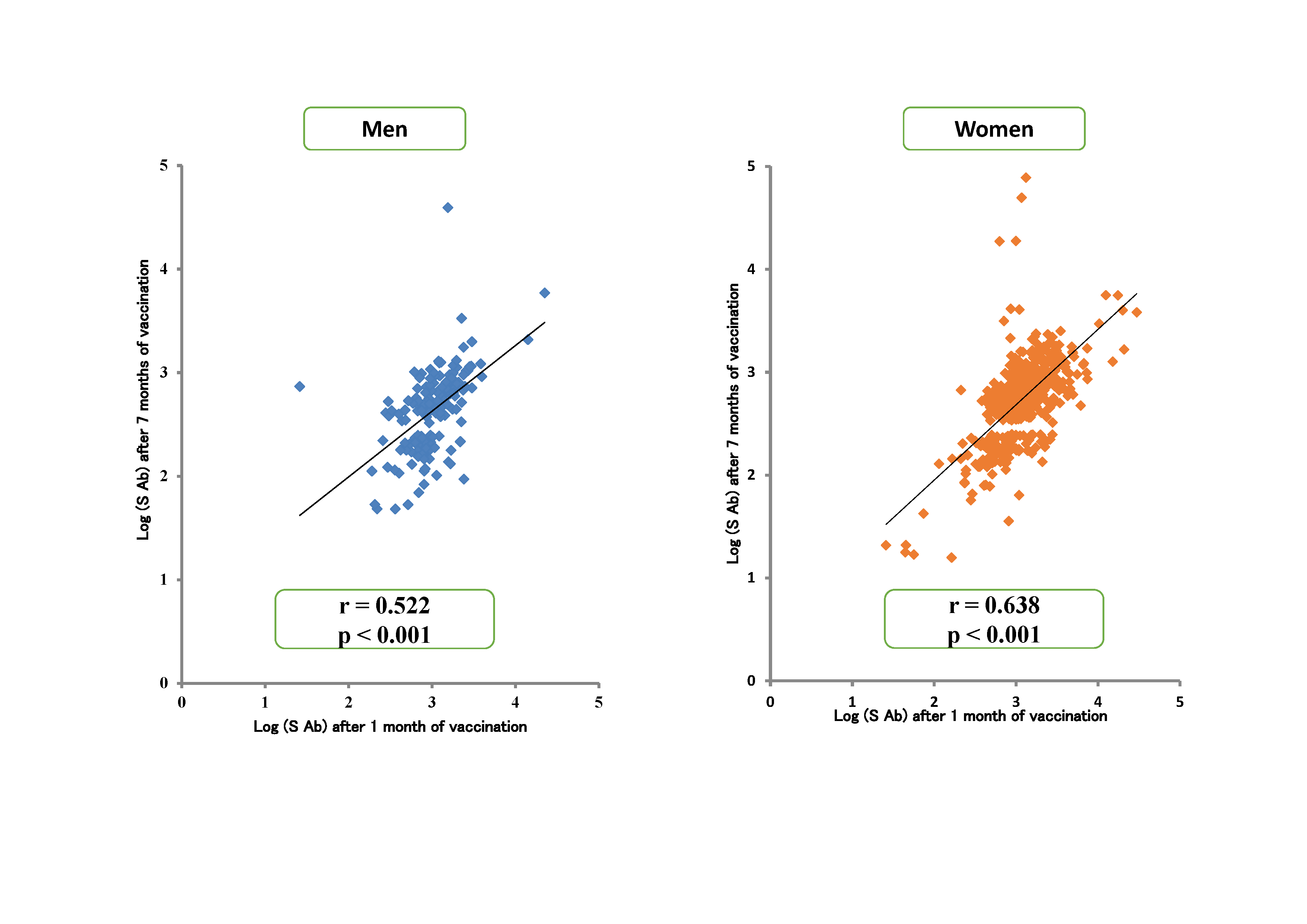

Supplement: Supplementary file 2 — Additional file 2: Figure S2. The correlation between the logarithmic values of anti-spike antibody titres [log (S Ab)] at one and seven months after vaccination was positively significant, and no differences were observed between the sexes. [file 12879_2022_7695_MOESM2_ESM.tif]
